# Accuracy of Artificial Intelligence-Based Models Versus Conventional Scoring Systems (APACHE, SOFA, and SAPS) in Predicting Mortality Among ICU Patients: A Systematic Review and Meta-Analysis

**DOI:** 10.7759/cureus.109459

**Published:** 2026-05-22

**Authors:** Vimukta Pradhan, Himanshu Shekhar, Punam Kumari Munda, Ashutosh Kumar Tiwari, Sneha Jha, Pratibha Rai

**Affiliations:** 1 General Medicine, Mahatma Gandhi Memorial Medical College and Hospital, Jamshedpur, IND; 2 Urology, All India Institute of Medical Sciences, Deoghar, IND; 3 Urology, Institute of Postgraduate Medical Education and Research (IPGMER), Kolkata, IND; 4 Surgical Gastroenterology, All India Institute of Medical Sciences, Deoghar, IND; 5 Cardiology, Institute of Medical Sciences, Banaras Hindu University, Varanasi, IND

**Keywords:** acute physiology and chronic health evaluation (apache), apache-ii score, apache iv, artificial intelligence (ai), in hospital mortality, intensive care unit, machine learning (ml), sequential organ failure assessment (sofa), simplified acute physiology score (saps) ii, systematic review and meta analysis

## Abstract

Accurate prediction of mortality in critically ill patients admitted to the ICU is essential for clinical decision-making and resource allocation. Conventional scoring systems such as Acute Physiology and Chronic Health Evaluation (APACHE), Sequential Organ Failure Assessment (SOFA), and Simplified Acute Physiology Score (SAPS) are widely used but are limited by their static structure and linear assumptions. Artificial intelligence (AI)-based models offer more flexible, data-driven approaches; however, their comparative performance remains uncertain. This systematic review evaluated the performance of AI-based models compared with conventional ICU scoring systems for predicting in-hospital mortality.

A systematic search of PubMed, the Excerpta Medica database (Embase), Web of Science, and Scopus was conducted from January 2015 to August 2025. Based on predefined eligibility criteria, studies comparing AI-based models with conventional scoring systems and reporting performance metrics such as area under the receiver operating characteristic curve (AUC), sensitivity, or specificity were included. Risk of bias was assessed using the Prediction model Risk Of Bias ASsessment Tool (PROBAST; Cochrane Prognosis Methods Group and the PROBAST Steering Group, University of Bristol, Bristol, United Kingdom, and collaborating international experts under the Cochrane Collaboration). A descriptive synthesis of discriminative performance was performed, along with a quantitative diagnostic test accuracy synthesis using a bivariate random-effects (Reitsma) model. Exploratory subgroup analyses using inverse variance-weighted random-effects meta-analysis with the DerSimonian-Laird estimator were conducted based on model type, dataset characteristics, and temporal modeling.

Ten studies involving approximately 500,000 ICU admissions were included. AI-based models demonstrated higher discriminative performance than conventional scoring systems, with reported AUC values ranging from 0.83 to 0.97 and ΔAUC ranging from 0.04 to 0.19. Eight studies contributed to the quantitative diagnostic test accuracy synthesis, yielding a pooled sensitivity of 0.845 (95% CI: 0.815-0.871) and a pooled specificity of 0.791 (95% CI: 0.728-0.843). Subgroup analyses demonstrated progressively higher pooled AUC values among tree-based/ensemble and deep learning models compared with classical machine learning (ML) approaches. Temporal and longitudinal models demonstrated pooled performance comparable to static variable-based models, while single-center cohorts demonstrated higher pooled AUC values than multicenter datasets. Calibration reporting was heterogeneous and not suitable for quantitative synthesis.

Overall, AI-based models show improved discriminative performance for mortality prediction in critically ill patients; however, substantial heterogeneity in study design, validation methodology, and reporting standards highlights the need for further external validation before routine clinical implementation.

## Introduction and background

Decisions regarding the prognosis of a critically ill patient in an ICU remain a major challenge, often requiring rapid and repeated assessment in daily practice. Clinicians are often required to estimate prognosis much before the complete clinical picture has evolved, making early assessment of mortality risk both essential and inherently uncertain. For decades, clinicians have relied largely on their own individual experience and level of expertise for assessment of risk, guiding treatment decisions, and prioritizing resource allocation [1,2]. This often led to variability in patient management and risk of death prediction [3]. Therefore, there was a need for more objective and consistent approaches that could reliably predict patient outcomes and be applied reliably across different clinical settings. In response, multiple scoring systems such as the Acute Physiology and Chronic Health Evaluation (APACHE), Sequential Organ Failure Assessment (SOFA), and Simplified Acute Physiology Score (SAPS) have been developed over time to standardize assessment and guide clinical decision-making in day-to-day life [3,4]. These conventional scoring systems are based mainly on physiological and biochemical parameters that are usually recorded within the first 24 hours of hospital admission and provide a structured severity of illness and, to a large extent, act as a supplementary tool in predicting the end outcomes [5-7].

Despite the widespread application of these conventional models over the years, there are various limitations that affect their accuracy in mortality prediction. Their performance varies significantly across different population subgroups and healthcare settings [7,8]. Also, they are based on relatively fixed sets of clinical or laboratory parameters and assume a linear relationship between predictors and outcomes [8,9]. In the real-world ICU settings, however, patient trajectories are rarely linear [4]. Clinical status of the patient changes rapidly with ongoing intervention, and interactions between the physiological and biochemical parameters are far more complex. As a result, traditionally used conventional scoring systems require periodic recalibration and may not always reflect the dynamic nature of critical illness and, subsequently, may lag in accurate mortality prediction [10,11].

With the evolution of electronic health records (EHRs) and greater availability of real-time monitoring data, new opportunities have emerged for more dynamic approaches to mortality prediction. The growing application of artificial intelligence (AI) and machine learning (ML) has ushered in a new generation of predictive modeling in critical care [9,10]. Unlike traditional approaches, these models not only handle high-dimensional data but also identify different patterns that might not be evident with standard statistical techniques [12,13]. Classical machine learning models, such as logistic regression and support vector machines (SVMs), primarily rely on predefined relationships between variables, whereas tree-based and boosting methods, including random forests and gradient boosting frameworks, are better aligned with modeling complex nonlinear interactions and subtle changes in biochemical parameters. In contrast, deep learning architectures, including neural networks and long short-term memory (LSTM)-based models, are advanced models capable of learning high-dimensional temporal and sequential patterns from time-series clinical data [14-16]. Studies utilizing large databases such as Medical Information Mart for Intensive Care (MIMIC-III), the electronic ICU (eICU) collaborative research database, and institutional EHR repositories have frequently reported that AI-based models demonstrate superior predictive accuracy in comparison with conventional ICU scoring systems [15-17]. The inherent adaptability of AI allows continuous recalibration, thereby enhancing model generalizability and sustaining predictive reliability over time.

A large number of studies have explored the utility of AI-based models for predicting in-hospital mortality, many of which have reported better discriminatory performance compared with conventional scoring systems, particularly when large datasets are used. Model performance in prediction research depends on discrimination and calibration, which reflect agreement between predicted and observed outcomes. Previous studies have shown that conventional scores like APACHE and SAPS II demonstrate moderate discrimination, but their performance often declines when applied to external or specialized cohorts [9-11,18]. However, the evidence is not uniformly consistent. Differences in study design, sample size, model development, and validation strategies make direct comparison across studies challenging [18]. In addition, many studies provide limited methodological details, particularly regarding internal validation and handling of overfitting. Reporting standards are often inconsistent, with limited external validation. Although several prior literature reviews have explored this field, important gaps still remain. Many previous studies were conducted before the widespread adoption of deep learning and ensemble methods. Many previous reviews often lacked a structured assessment of methodological quality using tools such as the Prediction model Risk Of Bias ASsessment Tool (PROBAST; Cochrane Prognosis Methods Group and the PROBAST Steering Group, University of Bristol, Bristol, United Kingdom, and collaborating international experts under the Cochrane Collaboration), which is now considered standard for evaluating prediction models [19]. Barboi et al. (2022), in a recent systematic review, have highlighted concerns regarding limited generalizability and inconsistent reporting across studies [19]. Given the rapid pace of development in this domain, an updated and methodologically robust synthesis of the available evidence is needed.

Therefore, the present systematic review and meta-analysis was undertaken to compare the performance of AI-based models with conventional scoring systems, including APACHE, SOFA, and SAPS, in predicting in-hospital mortality among adult ICU patients. By integrating currently available evidence, this study aims to clarify whether newer data-driven approaches provide meaningful advantages over established clinical prediction tools.

A version of this manuscript was previously published as a preprint on the medRxiv server on January 19, 2026.

## Review

Methodology

Study Design and Registration

This systematic review and meta-analysis was conducted in accordance with the Preferred Reporting Items for Systematic Reviews and Meta-Analyses (PRISMA 2020) [20].

The review protocol was registered prospectively with the International Prospective Register of Systematic Reviews (PROSPERO) under registration number CRD420251168073 [21].

Objectives 

The primary objective of this systematic review and meta-analysis was to compare the predictive performance of AI-based models with conventional scoring systems (APACHE II/IV, SOFA, and SAPS) for predicting mortality risk among adult ICU patients.

Secondary objectives included evaluating model discrimination using metrics such as area under the receiver operating characteristic curve (AUC); assessing methodological quality and risk of bias using the PROBAST tool; and exploring potential sources of heterogeneity through subgroup analyses based on AI model type, dataset characteristics (multicenter vs. single-center), and use of temporal modeling.

Eligibility Criteria

Eligibility criteria were defined according to the Population, Intervention, Comparison, Outcomes, and Study design (PICOS) framework, consistent with the study registration in PROSPERO; Population (P): Adult patients (≥18 years) admitted to medical, surgical, or mixed ICUs, Intervention (I): AI-based or ML-based models developed or validated for predicting mortality, Comparator (C): Conventional scoring systems, including APACHE II/IV, SOFA, or SAPS, were assessed within the same study cohort, Outcomes (O): Primary outcome: Predictive performance for in-hospital mortality, primarily assessed using discrimination metrics such as AUC. Where in-hospital mortality was not reported, ICU mortality was considered a surrogate outcome. Secondary outcomes: Additional performance measures, including sensitivity, specificity, and contingency table-derived diagnostic performance measures where available, along with subgroup analyses based on AI model type, dataset characteristics (multicenter vs. single-center), and use of temporal modeling. Studies reporting in-hospital mortality or ICU mortality were included. These outcomes were extracted and analyzed separately, and differences were considered during data synthesis. Study design (S): Retrospective or prospective cohort studies directly comparing AI-based models with conventional scoring systems and reporting outcome prediction performance metrics were eligible.

Inclusion Criteria

Studies were included if they involved adult critically ill patients (≥18 years) admitted to the ICU; evaluated or validated AI or ML models for predicting in-hospital or ICU mortality; and included a comparator, a conventional scoring system such as APACHE II/IV, SOFA, or SAPS. Eligible studies were required to report model performance using discrimination metrics, including the AUC, or provide sufficient information, including sensitivity, specificity, sample size, prevalence, or contingency data, to permit reconstruction of 2×2 contingency tables for diagnostic test accuracy analyses. Only peer-reviewed studies published in the English language were included.

Exclusion Criteria

Studies were excluded if they included pediatric populations (<18 years) or animal models, used simulated or non-clinical datasets, did not report mortality as an outcome, lacked a comparator conventional scoring system, or failed to provide sufficient data on predictive performance. Studies including mixed adult and pediatric populations were excluded to maintain population homogeneity. Reviews, commentaries, study protocols, conference abstracts, and other non-peer-reviewed publications were also excluded.

Search Strategy

A comprehensive literature search was conducted across PubMed, Excerpta Medica database (Embase), Scopus, and Web of Science for studies published between January 2015 and August 2025.

Both controlled vocabulary (MeSH terms) and free-text keywords were used, combined with Boolean operators to identify relevant studies on ICU mortality, AI, and conventional scoring systems.

The detailed PubMed search strategy was (“Intensive Care Units” [MeSH] OR “critical illness” OR “ICU”) AND (“mortality” OR “in-hospital mortality” OR “death”) AND (“artificial intelligence” OR “machine learning” OR “deep learning” OR “neural network” OR “predictive model”) AND (“APACHE” OR “SOFA” OR “SAPS” OR “scoring system”).

Equivalent search strategies, adapted to the indexing terms and syntax of each database, were applied to Embase, Scopus, and Web of Science. The complete search strategies for all databases are provided in Appendix A. The reference sections of all included articles were manually examined to locate any additional studies meeting the inclusion criteria. No additional eligible studies were identified through reference list screening. The final literature search was conducted on 30^th^ November 2025. Only peer-reviewed studies published in the English language were included.

Study Selection

All records identified through database searching were imported into reference management software, and duplicate entries were removed. The remaining records underwent title and abstract screening for eligibility. Title and abstract screening, followed by full-text assessment of potentially relevant articles, were performed independently and in duplicate by two reviewers. Full-text articles were evaluated against the predefined inclusion and exclusion criteria. Discrepancies at any stage were resolved through discussion and consensus; if disagreement persisted, a third reviewer was consulted for arbitration. Reasons for exclusion at the full-text stage were documented. The study selection process was conducted in accordance with PRISMA guidelines and is summarized in the PRISMA flow diagram.

Data Extraction

Two independent reviewers extracted data on study characteristics, cohort details, AI model type, comparator scoring system, validation method, sample size, and performance metrics (AUC, sensitivity, specificity). Discrepancies between reviewers were resolved through discussion and consensus; when agreement could not be reached, a third reviewer provided arbitration. 

A standardized data extraction form was developed and piloted before formal data extraction. Extracted variables included study characteristics, study design, patient population, ICU setting, sample size, AI model type, comparator scoring system, validation strategy, mortality outcome definition, contingency table data, sensitivity, specificity, AUC, calibration measures, and other reported performance metrics.

Where available, true positives (TP), false positives (FP), false negatives (FN), and true negatives (TN) were extracted directly from included studies. For studies not directly reporting contingency data, 2×2 contingency tables were reconstructed, where feasible, using reported sensitivity, specificity, total sample size, and prevalence/event rate. Reconstruction was performed using the following standard formulae: $TP = \mathrm{Sensitivity} \times \text{Number of deaths}$, $TN = \mathrm{Specificity} \times \text{Number of survivors}$, $FN = \text{Number of deaths} - TP$, $FP = \text{Number of survivors} - TN$, where $\text{Number of deaths} = \text{Mortality rate} \times \text{Total sample size}$, and $\text{Number of survivors} = \text{Total sample size} - \text{Number of deaths}$.

Reconstruction assumed internal consistency between reported diagnostic performance measures and study population characteristics. Studies with insufficient information for reliable reconstruction were excluded from diagnostic test accuracy synthesis. Calibration measures, including calibration plots, Brier score, expected calibration error, and Hosmer-Lemeshow statistics, were extracted where reported; however, due to substantial heterogeneity in reporting methods, quantitative synthesis of calibration performance was not performed.

Quality Assessment

The risk of bias, methodological quality, and applicability of the included studies were assessed using PROBAST [22]. Risk of bias assessment was performed independently by two reviewers, with discrepancies resolved through discussion and, where necessary, consultation with a third reviewer. This tool assesses four domains: participants, predictors, outcomes, and analysis. PROBAST was selected because it is specifically designed for evaluating prediction model development and validation studies, including those employing ML and deep learning architectures.

Statistical Analysis

Statistical analyses were performed using Review Manager (RevMan) version 5.4 (The Cochrane Collaboration, London, UK) and R software (metaDTA package; The R Core Team, R Foundation for Statistical Computing, Vienna, Austria).

For studies reporting complete or derivable contingency data, quantitative diagnostic test accuracy meta-analysis of sensitivity and specificity was performed using a bivariate random-effects model based on the Reitsma method with 95% confidence intervals. Hierarchical summary receiver operating characteristic (HSROC) curves with 95% confidence and prediction regions were generated to evaluate overall diagnostic discrimination and between-study variability. Heterogeneity between studies within the bivariate model was assessed using variance components and random-effects correlation parameters. In addition, complementary univariate random-effects analyses using restricted maximum likelihood (REML) estimation were performed to derive conventional heterogeneity statistics, including Cochran’s Q, I², and τ² for pooled sensitivity and specificity analyses.

Meta-analysis and subgroup analyses based on AI model type, ICU setting, and geographic region, validation method, and comparator scoring system were prespecified in the PROSPERO-registered study protocol; however, their implementation was limited by substantial heterogeneity in study designs, inconsistent reporting of performance metrics, and variability in AI model architectures. Accordingly, exploratory subgroup analyses based on ML model type, dataset characteristics (multicenter vs. single-center), and temporal modeling approach were performed using inverse variance-weighted random-effects meta-analysis with the DerSimonian-Laird estimator to further explore potential sources of heterogeneity across studies. Pooled AUC values with corresponding 95% confidence intervals were calculated for each subgroup.

Risk of bias was assessed using the PROBAST tool for all included studies. Formal sensitivity analyses stratified by risk-of-bias category were not performed because of substantial methodological heterogeneity, variability in study design, and overlap of risk-of-bias concerns across included studies. Instead, the potential influence of methodological limitations, particularly within the analysis domain, including risks related to overfitting, retrospective model development, and limited external validation, was considered qualitatively during interpretation of the pooled findings and overall strength of evidence. Formal assessment of publication bias was not performed due to the limited number of studies; however, potential bias was considered qualitatively, recognizing that studies reporting favorable performance of AI models may be more likely to be published.

Data Sources and Final Synthesis

The final synthesis included 10 studies that met the predefined eligibility criteria, representing diverse ICU populations, AI model architectures, and clinical settings. Eight studies provided directly reported or derivable contingency data and were included in the diagnostic test accuracy meta-analysis, while all included studies contributed AUC-based performance data for qualitative and subgroup analyses. Study findings were summarized in structured tables describing study characteristics, AI model types, comparator scoring systems, validation approaches, outcome definitions, and reported predictive performance metrics. Quantitative findings were presented using forest plots for pooled sensitivity and specificity, along with HSROC curves with corresponding confidence and prediction regions.

Data Availability

The data supporting the findings of this systematic review and meta-analysis were derived from previously published studies indexed in publicly accessible academic databases. Extracted study-level data, pooled analyses, subgroup analyses, reconstructed contingency data, and quantitative synthesis results are presented within the main manuscript tables, figures, and appendices. Complete electronic search strategies for all databases searched, detailed PROBAST frameworks, a standardized data extraction form, annotated R statistical analysis scripts used for diagnostic test accuracy synthesis and subgroup analyses, and a comparison table between the prespecified PROSPERO protocol and final review methodology are provided in Appendices A-E, respectively.

Results

The initial search across four electronic databases, supplemented by manual reference screening, identified a total of 196 records. After removal of 68 duplicates, 128 titles and abstracts were screened, of which 96 articles were selected for full-text assessment. Of these, 83 full-text articles were successfully retrieved and reviewed in detail. A total of 73 studies were excluded due to reasons such as incomplete reporting of performance metrics, absence of AI-based models, inclusion of pediatric populations, or lack of relevant outcomes. Ultimately, 10 studies were selected for inclusion in the qualitative synthesis based on the predefined eligibility criteria [3, 5, 6, 8, 10, 12-16]. Among the selected studies, eight studies provided contingency data and were included in the diagnostic test accuracy meta-analysis of sensitivity and specificity [3, 5, 6, 8, 12, 13, 15, 16].

**Figure 1:**
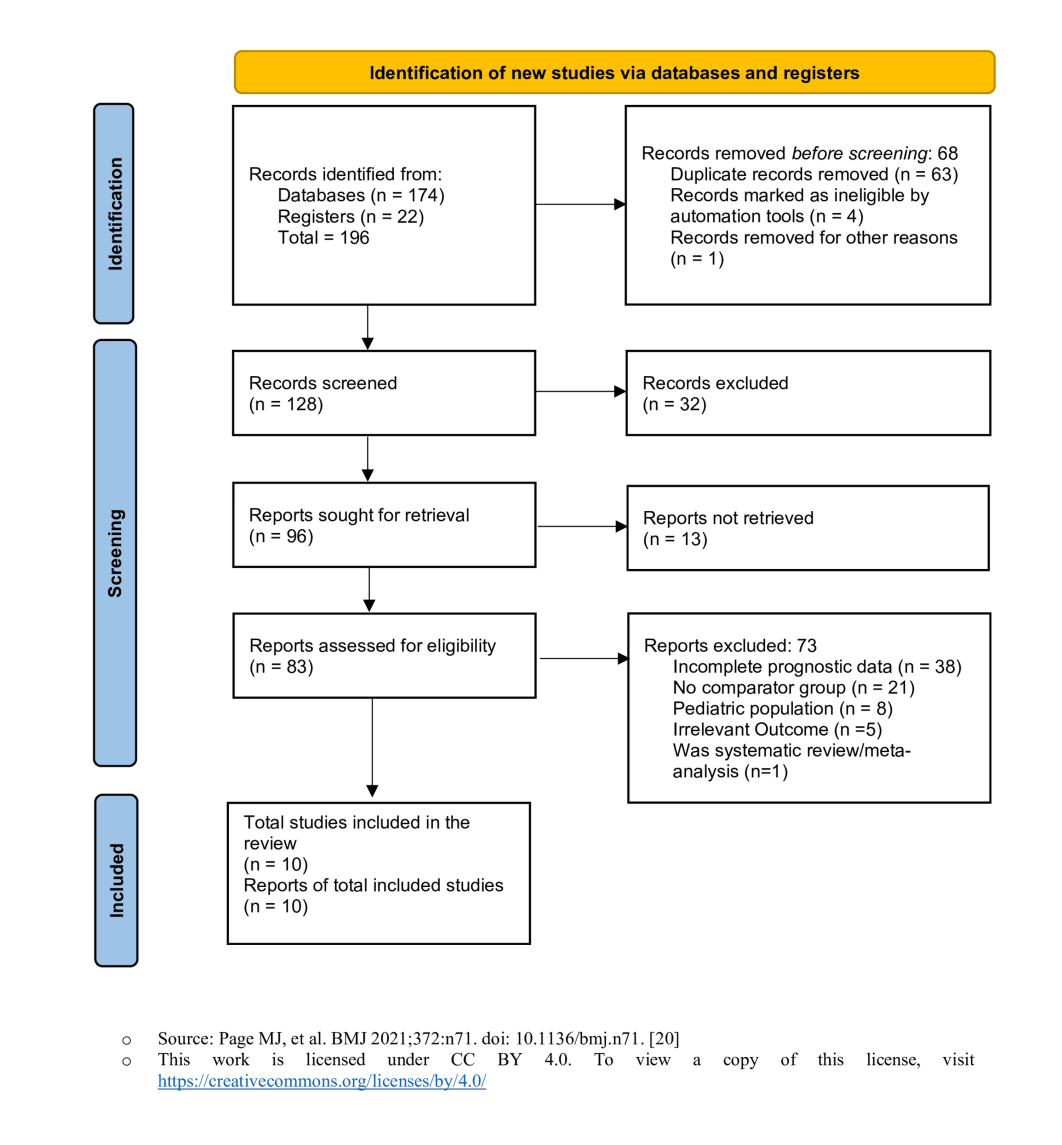
The PRISMA 2020 flow diagram depicting the identification, screening, eligibility, and inclusion of studies in the systematic review and meta-analysis evaluating AI-based models versus conventional scoring systems (Acute Physiology and Chronic Health Evaluation (APACHE), Sequential Organ Failure Assessment (SOFA), and Simplified Acute Physiology Score (SAPS)) for mortality prediction. Reference: [20]

The PRISMA 2020 flow diagram (Figure 1) illustrates the study selection process. The included studies, published between January 2015 and August 2025, collectively analyzed data from about 0.5 million hospital admissions and ICU admissions across diverse geographic regions, including North America, Europe, and Asia.

The included studies evaluated a range of AI-based models, including gradient boosting machines, random forests, ensemble methods, and deep neural networks, developed using routinely collected clinical data, including EHRs. All included studies compared AI-based predictive models with conventional scoring systems (APACHE II/IV, SOFA, or SAPS II). Most studies reported either internal or external validation, with several utilizing multicenter datasets. Detailed study characteristics are summarized in Table 1.

**Table 1:** Baseline characteristics and performance of included studies evaluating AI-based models versus conventional scoring systems for mortality prediction. The table summarizes study design, dataset type, sample size, AI model used, comparator scoring systems, and main findings reported in each study. AI: artificial intelligence; ICU: intensive care unit; APACHE: Acute Physiology and Chronic Health Evaluation; SOFA: Sequential Organ Failure Assessment; SAPS: Simplified Acute Physiology Score; ML: machine learning; AUC: area under the receiver operating characteristic curve; CI: confidence interval; LR: logistic regression; LSTM: long short-term memory; RF: random forest; XGB: extreme gradient boosting; MLP: multilayer perceptron; SVM: support vector machine; DT: decision tree; CART: classification and regression tree; CKD: chronic kidney disease; EHR: electronic health record.

Study (year)	County/Dataset	Sample size/Outcome	AI model(s) used	Comparator(s)	Main results	Accuracy (AI/Traditional)
Jeon ET et al., 2023 [3]	South Korea; single-center ICU dataset	n = 816 ICU patients with severe pneumonia, deaths = 223 (27.3%).	Logistic regression (L2), LightGBM (GBDT), MLP	SAPS II, APACHE II, SOFA	ML models significantly outperformed conventional scores.	AUC: Logistic regression ML 0.820 - 0.838, LightGBM 0.827, MLP 0.838. vs. SAPS II 0.650 (0.584 - 0.716), (AUROC presented)
Mirzakhani F et al., 2022 [5]	Iran single-center ICU dataset	n = 840 (mortality ~33%)	MLP (neural network), CART/decision tree variants	SOFA, SAPS II, APACHE II, APACHE IV	MLP NN showed the best performance. External validation discussed.	AUC: MLP 0.841 vs. CART 0.800.
Lin X et al., 2024 [6]	USA MIMIC-IV database	Total n = 1,984 MI patients (derivation n = 1,389; validation n = 595).	XGBoost, Random Decision Forest (RDF), and nomogram (ML-based)	SOFA	An ML-based nomogram outperformed SOFA in mortality prediction.	AUC: Nomogram 0.835 vs SOFA 0.735; Accuracy: 0.914 vs 0.913
Deng Y et al., 2025 [8]	USA MIMIC-IV dataset	n = 1,336 ICU patients.	LSTM (hourly time-series representations)	Conventional LR implementations of APACHE II, SAPS II, SOFA (LR using the same inputs)	LSTM-based models outperformed corresponding logistic regression models across all scoring systems.	AUC reported: LSTM 0.861–0.898 vs. LR 0.708–0.777.
Lim L et al., 2024 [10]	South Korean development cohort with international external validation datasets	Large cohorts—internal derivation/validation numbers reported by paper—derivation n = 1,389; validation n = 595 for a MIMIC-IV-based subanalysis noted, and it contains multiple cohort counts and site-specific Ns.	Ensemble ML (real-time EHR variables)	APACHE II, SAPS II, SOFA	The ensemble model demonstrated superior performance with strong internal and external validation.	AUC: nomogram ~0.835 (subanalysis); SOFA ~0.735.
Huang T et al.,2023 [12]	USA MIMIC-IV and validation dataset USA eICU-CRD	Total n = 5,052 (training n = 2,638; validation n = 2,414); In-hospital mortality: 24.8% (training), 21.7% (validation)	LR, RF, DT, LightGBM, XGBoost, Ensemble (RF+LightGBM+XGBoost)	Conventional scoring systems (SOFA, OASIS, APS III used as predictors/features, not direct comparators)	The ensemble model (RF+LightGBM+XGBoost) achieved the best performance among ML models.	AUC: ensemble model ≈ 0.92 (training) and ≈ 0.93 (validation)
Pang K et al., 2022 [13]	USA (MIMIC-IV database)	MIMIC-IV full extraction: 67,748 patients, sample n = 14,110 (7,055 deceased + 7,055 survivors) for modeling; training n = 9,877; validation n = 4,233.	XGBoost, SVM, LR, Decision Tree (multiple ML models)	APACHE II, SAPS II (and others depending on analysis)	XGBoost performed best; ML models outperformed conventional scores.	AUC: XGBoost 0.918; LR 0.872; SVM 0.872; DT 0.852
Choi MH et al.,2022 [14]	South Korea Two-center hospital-based cohort	Total n = 85,146 (combined data from two hospitals, 2006–2020).	Multiple ML algorithms: KNN, DT, RF, XGBoost, LightGBM, SVM, ANN	APACHE, SOFA, SAPS (various scores depending on cohort)	ML models showed substantially higher AUROC compared with conventional scores, and external validation performance was lower.	Best ML AUROCs: 0.977 and 0.955 in best models; conventional scores lower; SAPS III 0.773, APACHE III 0.803
Li X et al., 2023 [15]	USA (MIMIC-IV database)	n = 8,527 CKD patients (from MIMIC-IV, 2008–2019); reported mortality and cohort descriptors	Multiple ML models (six models, XGBoost among the top)	SOFA, SAPS II (conventional severity scores included)	XGBoost had the highest AUC (0.860); ML models outperformed conventional scores.	AUC: XGBoost 0.860, SOFA AUC = 0.762, SAPS II AUC = 0.768
Selçuk M., 2022 [16]	Turkey single-center ICU cohort	n = 200 ICU sepsis patients (2015–2020).	Multiple ML classifiers (RF, XGB, MLP, etc.)	APACHE II, SAPS II, SOFA	ML classifiers outperformed conventional scoring systems in sepsis mortality prediction.	AUC (best ML) ≈ 0.85 APACHE II AUC: 0.82 SAPS II AUC: 0.78 SOFA AUC: 0.80

Risk of bias assessment

**Figure 2:**
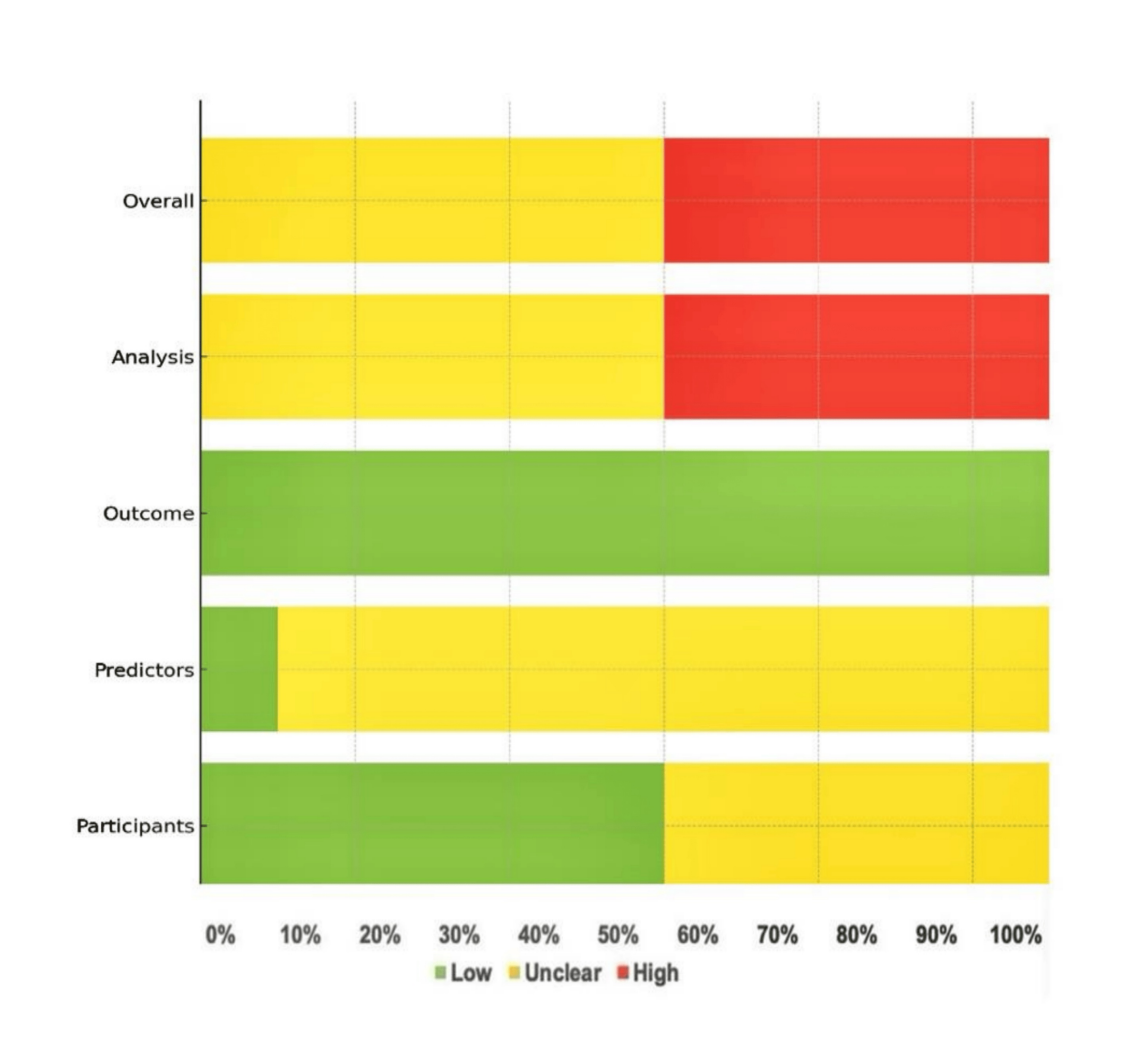
Summary of risk of bias assessment across included studies using the Prediction model Risk Of Bias ASsessment Tool (PROBAST). The proportions of studies rated as green (low), yellow (some risk/unclear), or red (high) risk of bias are shown for each of the domains: participants, predictors, outcome, analysis and overall assessment. [3,5,6,8,10,12-16]

Risk-of-bias assessment was conducted using PROBAST across the included primary studies [22]. Overall, most studies demonstrated low risk of bias across the participants, predictors, and outcome domains, with comparatively greater concerns identified within the analysis domain, although some variability was observed across studies. A summary of the risk-of-bias assessment across all included studies is presented in Figure 2.

The majority of the studies included clearly defined ICU populations derived from EHRs or established clinical databases, supporting low risk in the participants' domain. Predictors were generally measured before outcome determination and were broadly consistent with variables used in conventional scoring systems, resulting in low to moderate concern in the predictors' domain. Outcome assessment was based on objective mortality endpoints and was consistently defined across studies, indicating low risk in the outcome domain.

**Figure 3:**
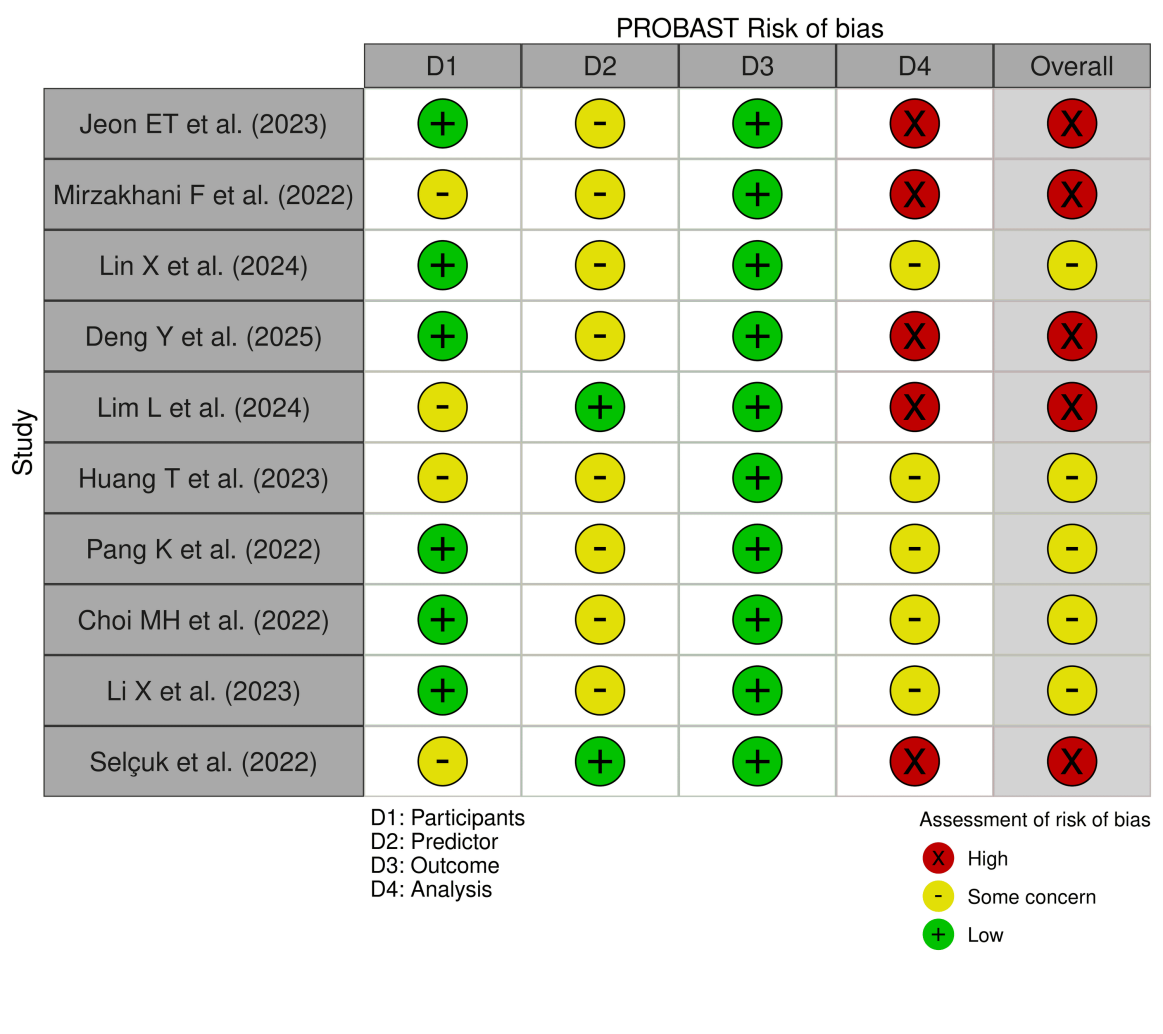
Risk of bias assessment of each included studies using the Prediction model Risk Of Bias ASsessment Tool (PROBAST). Domain-specific risk of bias assessment of each included study using the PROBAST tool. Each colored cell represents the risk of bias rating for that domain: low risk (green), some risk (yellow), and high risk (red), across participants, predictors, outcome, and analysis domains, with an overall assessment for each study [3,5,6,8,10,12-16].

The analysis domain represented the main area of methodological variability. Several studies, especially those using high-capacity learning architectures (Deng Y et al., Pang K et al., Choi MH et al., and Selçuk M et al.), do not fully report key aspects of model development, including internal validation methods, hyperparameter tuning, and calibration assessment [8,13,14,16]. In several studies, random data-splitting approaches were used, while temporally or externally validated cohorts were less frequently reported, which may increase the risk of overfitting and limit generalizability. Variability in reporting of model thresholds and performance metrics was also noted. The LSTM-based modeling study by Deng Y et al. (2025) exhibited high bias due to the absence of an explicit temporal validation design and limited reporting of calibration performance [8]. Study-level risk-of-bias assessments are summarized in Figure 3. Overall, these findings indicate variability in methodological quality, particularly within the analysis domain.

Applicability concerns were generally low, as all studies evaluated adult ICU populations using routinely collected clinical predictors and clinically relevant mortality outcomes aligned with the objectives of this review.

Discriminative performance across included studies

**Figure 4:**
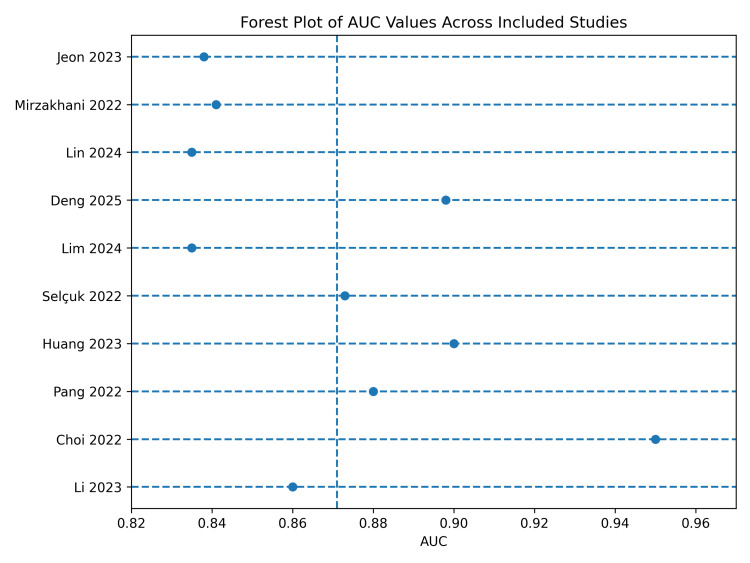
Forest plot of area under the curve (AUC) values comparing AI-based models for mortality prediction across the included studies. Each point represents the AUC of the best-performing AI-based model in each study. The dashed vertical line indicates the mean AUC (0.87), summarizing overall discriminative performance across studies [3,5,6,8,10,12-16].

This section summarizes the discriminative performance of AI-based models across included studies, primarily using AUC and related metrics. Across all 10 included studies, AI-based models consistently demonstrated high discriminative performance for mortality prediction [3,5,6,8,10,12-16]. Reported AUC values ranged from 0.83 to 0.95, with a mean AUC of 0.87. Most studies reported AUC values above 0.85, indicating consistent discriminative performance across diverse patient populations and clinical settings. A forest plot of the AUC of the best-performing AI-based model in each study is represented in Figure 4.

The highest AUC values were observed in studies using ensemble and advanced ML techniques, such as gradient boosting and deep learning models, while comparatively lower values were noted in smaller, single-center studies. Despite variations in study design, dataset size, and model architecture, all included studies reported AUC values exceeding 0.80, reflecting consistently good model performance

Comparative performance between AI-based models and conventional scoring systems

This section evaluates the comparative performance of AI-based models relative to conventional clinical scoring systems, emphasizing differences in discriminative accuracy across included studies. AI-based models consistently demonstrated higher AUC values than conventional scoring systems, including APACHE, SOFA, and SAPS, across most studies, supporting the robustness of this trend across diverse ICU populations and healthcare settings.

The reported AUC values for AI-based models ranged from 0.82 to 0.90, exceeding those of conventional scoring systems, including APACHE II (0.70-0.78), SOFA (0.68-0.75), and SAPS II (0.70-0.79). Across studies reporting the comparative performance, the difference in AUC (ΔAUC) between AI-based models and conventional scoring systems ranged from +0.04 to +0.19 [3, 5, 6, 8, 10, 12-16]. Except for the study by Mirzakhani et al. [5], all included studies demonstrated a ΔAUC greater than +0.10 in favor of AI-based models [3,5,6,8,10,12-16].

Given the substantial heterogeneity in study populations, model architectures, validation methodologies, and the limited availability of variance estimates, comparative findings were interpreted descriptively without formal statistical testing. Nevertheless, the included studies consistently demonstrated improved discriminative performance of AI-based models compared with conventional scoring systems. A summary of comparative AUC values across studies is presented in Table 2.

**Table 2:** Comparative discriminative performance of AI-based models and conventional scoring systems. The table summarizes the area under the receiver operating characteristic curve (AUC) values for the best-performing AI-based model and corresponding conventional scoring system in each included study, along with the difference in AUC (ΔAUC). Values are presented as reported in the original studies [3,5,6,8,10,12-16]. Abbreviations: SOFA, Sequential Organ Failure Assessment; APACHE II, Acute Physiology and Chronic Health Evaluation II

Study	AI Model (Best AUC)	Conventional Score (AUC)	ΔAUC
Jeon ET et al., 2023 [3]	0.838	0.650 (SAPS II)	+0.19
Mirzakhani F et al., 2022 [5]	0.84	0.80	+0.04
Lin X et al., 2024 [6]	0.835	0.735 (SOFA)	+0.10
Deng Y et al., 2025 [8]	0.898	0.777 (APACHE II)	+0.12
Lim L et al., 2024 [10]	0.835	0.735 (SOFA)	+0.10
Huang T et al., 2023 [12]	0.90	0.78	+0.12
Pang K et al., 2022 [13]	0.88	0.75	+0.13
Choi MH et al., 2022 [14]	0.95	0.80	+0.15
Li X et al., 2023 [15]	0.860	~0.75	+0.11
Selçuk M., 2022 [16]	0.873	~0.75	+0.12

Subgroup analysis

This section presents subgroup analyses exploring differences in predictive performance by ML model type, temporal modeling approach, and dataset characteristics. Subgroup analyses were performed using inverse variance-weighted random-effects meta-analysis with the DerSimonian-Laird estimator at the model/cohort level rather than the study level; therefore, several studies contributed more than one independently validated model or cohort to different subgroup syntheses. Cohorts included within each subgroup analysis were mutually exclusive. Studies with unclear subgroup classification or without extractable AUC and 95% confidence interval data were excluded from quantitative pooling. Formal statistical testing for subgroup differences was not performed because the analyses were exploratory, and substantial heterogeneity was observed across studies.

Model Type

Subgroup analysis based on ML model type demonstrated progressive improvement in pooled discriminative performance from classical ML models to advanced deep learning approaches. Classical ML models, including logistic regression, SVM, and K-nearest neighbors (KNN) algorithms, demonstrated the lowest pooled AUC of 0.78 (95% CI: 0.70-0.87). Tree-based and ensemble models, including random forest, XGBoost, and LightGBM, demonstrated better pooled performance with a pooled AUC of 0.86 (95% CI: 0.82-0.89). Deep learning and temporal models, including multilayer perceptron and LSTM frameworks, demonstrated the highest pooled AUC of 0.89 (95% CI: 0.85-0.93). Considerable heterogeneity was observed across all subgroup analyses (I² > 98%). Detailed results of the subgroup analysis based on the ML model type are presented in Table 3.

**Table 3:** Subgroup analysis of model performance based on machine learning model type. Pooled AUC values were calculated using inverse variance-weighted random-effects meta-analysis with the DerSimonian–Laird estimator. Subgroup analyses were performed at the model/cohort level; therefore, multiple independently validated models or cohorts from a single study could contribute to different subgroup syntheses. The reported AUC range represents the minimum and maximum AUC values observed within each subgroup. Considerable heterogeneity was observed across cohorts in all subgroup analyses. Abbreviations: AUC, area under the receiver operating characteristic curve; CI, confidence interval; I², inconsistency statistic; RF, random forest; SVM, support vector machine; KNN, k-nearest neighbors; XGBoost, extreme gradient boosting; LightGBM, light gradient boosting machine; MLP, multilayer perceptron; LSTM, long short-term memory.

Model Type	Included Models	Studies/Cohorts (n)	Reported AUC Range	Pooled AUC (95% CI)	I^2^
Classical machine learning	Logistic regression, SVM, KMN	5	0.64-0.87	0.78 (0.70-0.87)	98.7%
Tree-based/ensemble models	RF, XGBoost, LightGBM	8	0.59-0.97	0.86 (0.82-0.89)	99.4%
Deep learning/temporal models	MLP, LSTM, hybrid DL frameworks	5	0.84-0.96	0.89 (0.89-0.93)	99.7%

Dataset Characteristics

An exploratory subgroup analysis based on dataset characteristics, i.e., single-center vs. multi-center dataset, was performed using inverse-variance-weighted random-effects meta-analysis with the DerSimonian-Laird estimator. Multicenter datasets included MIMIC-IV and eICU-based cohorts and demonstrated a pooled AUC of 0.89 (95% CI: 0.88-0.90) across five cohorts, with reported AUC values ranging from 0.83 to 0.92. In contrast, single-center cohorts demonstrated a pooled AUC of 0.94 (95% CI: 0.93-0.95) across six cohorts, with reported AUC values ranging from 0.82 to 0.97. Multicenter cohorts were predominantly represented by externally validated heterogeneous ICU populations, whereas single-center cohorts mainly consisted of internally validated institutional datasets. Studies without extractable AUC or 95% confidence interval data were excluded from quantitative synthesis.

Temporal Modeling

Exploratory subgroup analysis based on the temporal modeling approach demonstrated relatively similar pooled discriminative performance between temporal/longitudinal and static variable-based models. Temporal or longitudinal models, including LSTM-based architectures, multilayer perceptron frameworks, and other sequential deep learning approaches, utilized serial or real-time physiological measurements and time-dependent clinical trajectories for prediction. In contrast, static variable-based models primarily relied on admission-based, baseline, or summarized clinical variables without incorporating temporal trends or repeated sequential data. Longitudinal models demonstrated a pooled AUC of 0.90 (95% CI: 0.86-0.94) across five cohorts, with reported AUC values ranging from 0.87 to 0.96. Static models demonstrated a marginally better pooled AUC of 0.91 (95% CI: 0.88-0.94) across six cohorts, with reported AUC values ranging from 0.83 to 0.97. A summary of subgroup analyses according to dataset characteristics and use of temporal modeling is presented in Table 4.

**Table 4:** Subgroup analysis of predictive performance according to dataset characteristics and temporal modeling approach. This table summarizes the discriminative performance of AI-based models across subgroups defined by dataset characteristics and temporal modeling approach. Multicenter datasets refer to studies utilizing large multi-institutional or public ICU databases, whereas single-center datasets represent institution-based cohorts. Temporal models incorporated longitudinal or sequential clinical data, while static models were based on admission or summarized clinical variables. Pooled AUC values were calculated using inverse variance-weighted random-effects meta-analysis with the DerSimonian–Laird estimator. Subgroup analyses were performed at the cohort/model level; therefore, multiple independently validated cohorts from a single study could contribute to different subgroup syntheses. The reported AUC range represents the minimum and maximum AUC values observed within each subgroup. Abbreviations: AUC, area under the receiver operating characteristic curve; CI, confidence interval; ICU, intensive care unit; LSTM, long short-term memory; MIMIC-IV, Medical Information Mart for Intensive Care IV; eICU-CRD, eICU Collaborative Research Database.

Subgroup Category	Subgroup	Number of Cohorts	Reported AUC Range	Weighted Pooled AUC (95% CI)	Key Observation
Dataset characteristics	Multicenter datasets (MIMIC-IV, eICU-CRD)	5	0.83-0.92	0.89 (0.88-0.90)	Included external validation and heterogeneous ICU populations.
	Single-center	6	0.82-0.97	0.94 (0.93-0.95)	Predominantly internally validated institutional cohorts.
Temporal modeling	Temporal/longitudinal models (e.g., LSTM)	5	0.87-0.96	0.90 (0.86-0.94)	Utilized sequential or real-time longitudinal physiological data and showed variable performance
	Static variable-based models	6	0.83-0.97	0.91 (0.88-0.94)	Primarily based on admission or summarized clinical variables

Prognostic accuracy of AI-based models: findings from quantitative synthesis

A diagnostic test accuracy meta-analysis was performed using a bivariate random-effects model based on the Reitsma method to quantitatively synthesize the discriminative performance of AI-based mortality prediction models across included ICU studies. Only studies reporting sufficient data for the extraction or reconstruction of 2×2 contingency tables were eligible for quantitative synthesis. Eight studies were included in the diagnostic test accuracy meta-analysis [3,5,6,8,12,13,15,16]. Study-level 2×2 contingency data, including TP, FN, FP, and TN, were extracted or reconstructed from the included studies for quantitative synthesis using standard statistical formulas. Reported sensitivity values ranged from 0.776 to 0.897, while specificity values ranged from 0.660 to 0.890 across the included studies. Sample sizes varied considerably, with Pang et al. contributing the largest cohort (n=4,233) and Selçuk et al. representing the smallest study population (n=200) [13,16]. Table 5 summarizes the studies included in the diagnostic test accuracy meta-analysis and their corresponding extracted or reconstructed 2×2 contingency table data and diagnostic performance measures.

**Table 5:** Study-level diagnostic performance data for studies included in the bivariate diagnostic test accuracy meta-analysis. The table summarizes the studies included in the diagnostic test accuracy meta-analysis and the extracted 2×2 contingency table data used for quantitative synthesis using the bivariate random-effects Reitsma model. Abbreviations: TP, true positive; FN, false negative; FP, false positive; TN, true negative; Sens, sensitivity; Spec, specificity; N represents the total study population.

Study/Year	TP	FN	FP	TN	Total N	Sensitivity	Specificity
Jeon et al., 2023 [3]	179	44	178	415	816	0.803	0.700
Mirzakhani F et al., 2022 [5]	282	51	141	366	840	0.847	0.722
Lin X et al., 2024 [6]	59	7	180	349	595	0.894	0.660
Deng Y et al., 2025 [8]	200	23	257	856	1336	0.897	0.769
Huang T et al., 2023 [12]	465	58	208	1683	2414	0.889	0.890
Pang K et al., 2022 [13]	1739	377	326	1791	4233	0.882	0.846
Li X et al., 2023 [15]	227	54	318	1106	1705	0.808	0.777
Selcuk M et al., 2022 [16]	52	15	15	118	200	0.776	0.887

**Figure 5:**
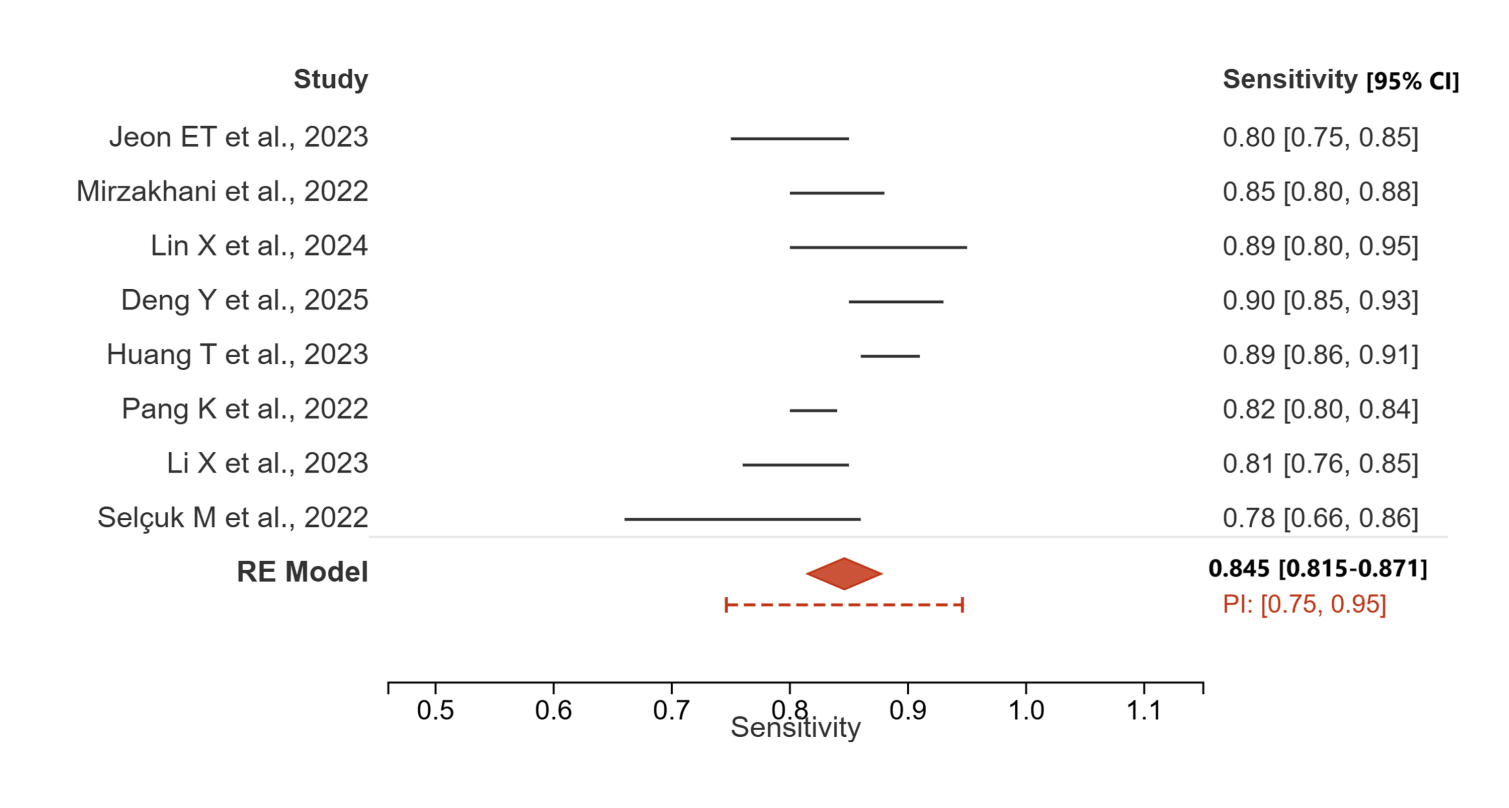
Forest plot demonstrating pooled sensitivity estimates of AI-based mortality prediction models across included studies using a random-effects meta-analysis model. The diamond represents the pooled sensitivity estimate, while the dashed line indicates the prediction interval (PI) [3,5,6,8,12,13,15,16].

**Figure 6:**
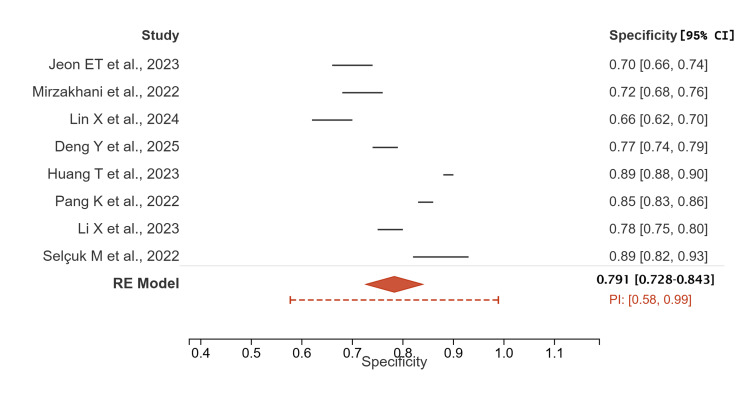
Forest plot demonstrating pooled specificity estimates of AI-based mortality prediction models across included studies using a random-effects (RE) meta-analysis model. The diamond represents the pooled specificity estimate, while the dashed line indicates the prediction interval (PI) [3,5,6,8,12,13,15,16].

Quantitative synthesis was performed using a bivariate random-effects model based on the Reitsma method across eight studies reporting sufficient diagnostic contingency data. The pooled sensitivity of AI-based mortality prediction models was 0.845 (95% CI: 0.815-0.871), while the pooled specificity was 0.791 (95% CI: 0.728-0.843). The pooled diagnostic odds ratio was 20.664 (95% CI: 13.586-31.429), indicating overall good discriminative performance across the included studies. Table 6 summarizes the pooled diagnostic performance estimates derived from the bivariate random-effects meta-analysis, including pooled sensitivity, specificity, and diagnostic odds ratio with corresponding 95% confidence intervals. Study-level sensitivity estimates ranged from 0.776 to 0.897, whereas specificity estimates ranged from 0.660 to 0.890. Prediction intervals were wider for specificity than for sensitivity, suggesting comparatively greater between-study variability in specificity estimates. Forest plots demonstrating pooled sensitivity and specificity derived from the random-effects model are presented in Figures 5-6, respectively.

**Table 6:** Pooled diagnostic accuracy estimates of AI-based models for mortality prediction using the Reitsma bivariate random-effects model. Pooled diagnostic accuracy estimates derived from the bivariate random-effects (Reitsma) model for studies included in the quantitative synthesis. Abbreviations: CI, confidence interval; DOR, diagnostic odds ratio.

Metric	Pooled Estimate	95%CI
Sensitivity	0.845	0.815-0.871
Specificity	0.791	0.728-0.843
Diagnostic odds ratio	20.664	13.586-31.429

Heterogeneity assessment using the Reitsma bivariate random-effects model demonstrated a low random-effects correlation between sensitivity and specificity (r = 0.087), suggesting the absence of a significant threshold effect and relatively stable diagnostic discrimination across the included studies. The between-study variance component for specificity (σα = 0.256) was substantially greater than that for sensitivity (σθ = 0.054), indicating comparatively greater heterogeneity in specificity estimates than in sensitivity estimates. Table 7 summarizes the findings of heterogeneity and random-effect parameters, derived from the Reitsma bivariate model. 

**Table 7:** Heterogeneity and random-effects parameters derived from the Rietma bivariate model. Parameters derived from the bivariate random-effects (Reitsma) model used for diagnostic accuracy synthesis. Random-effects correlation reflects the association between sensitivity and specificity across studies. Abbreviations: HSROC, hierarchical summary receiver operating characteristic; θ, overall HSROC accuracy parameter; σθ, variance component for sensitivity; σα, variance component for specificity.

Parameter	Estimate
Random-effects correlation	0.087
θ (overall HSROC accuracy parameter)	0.736
σθ (variance component for sensitivity)	0.054
σα (variance component for specificity)	0.256

Complementary REML-based random-effects analyses also demonstrated substantial inter-study heterogeneity. For pooled sensitivity analysis, Cochran’s Q was 34.11 with an I² of 79.5%, indicating substantial heterogeneity across studies. However, the corresponding between-study variance estimate for sensitivity was relatively low (τ² = 0.0014), suggesting modest absolute dispersion in pooled sensitivity estimates despite the high relative heterogeneity. Heterogeneity was very high for pooled specificity, with a Cochran’s Q of 311.42 and an I² of 97.8%, indicating considerable between-study variability in specificity estimates. In addition, the larger between-study variance for specificity (τ² = 0.0063) demonstrated greater absolute dispersion in study-level specificity values across the included studies. The findings are shown in Table 8.

**Table 8:** Conventional heterogeneity statistics derived from REML random-effects meta-analysis. The table summarizes conventional heterogeneity statistics derived from separate univariate random-effects analyses using REML estimation. Abbreviations: REML, restricted maximum likelihood; Q, Cochran’s Q statistic; df, degrees of freedom; I², inconsistency statistic; τ², between-study variance.

Parameter	Sensitivity	Specificity
Cochran’s Q	34.11	311.42
Degrees of freedom (df)	7	7
I^2^	79.5%	97.8%
τ^2^	0.0014	0.0063

**Figure 7:**
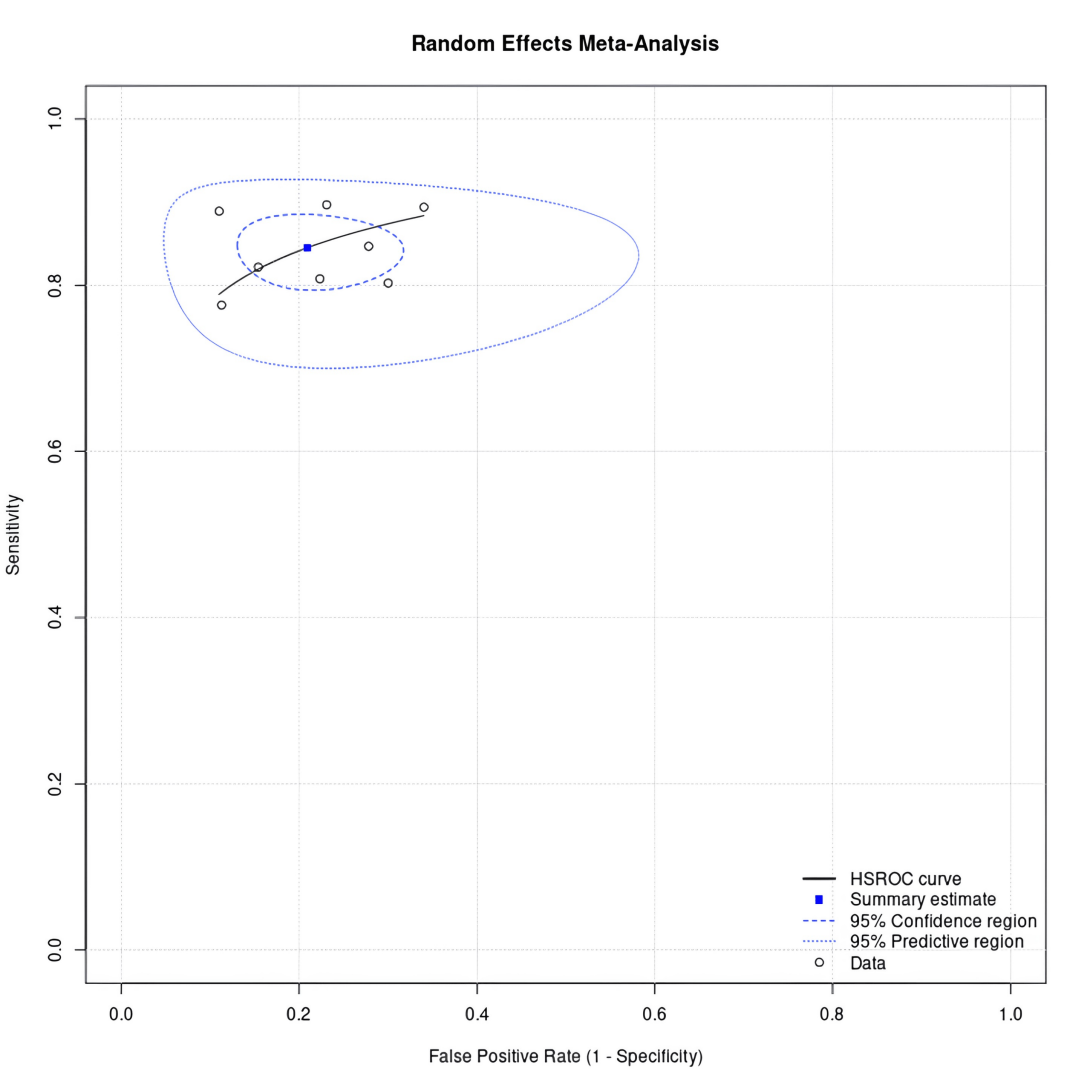
Hierarchical summary receiver operating characteristic (HSROC) curve of AI-based mortality prediction models. The HSROC curve demonstrating the overall diagnostic performance of AI-based mortality prediction models across included studies using the Reitsma bivariate random-effects model. Open circles represent individual studies, the blue square represents the pooled summary estimate, the inner dashed ellipse indicates the 95% confidence region, and the outer dashed ellipse represents the 95% prediction region.

The HSROC curve demonstrated overall good discriminative performance of AI-based mortality prediction models across included studies (Figure 7). Individual study estimates were clustered around the pooled summary operating point, corresponding to a pooled sensitivity of 0.845 and a pooled specificity of 0.791. The overall HSROC accuracy parameter (θ) derived from the Reitsma bivariate random-effects model was 0.736, supporting favorable overall diagnostic discrimination of AI-based mortality prediction models across heterogeneous ICU populations. The relatively compact confidence region suggested acceptable precision around the pooled estimate, whereas the broader prediction region reflected underlying between-study heterogeneity, particularly for specificity estimates. The low random-effects correlation observed in the Reitsma model further supported the absence of a substantial threshold effect across included studies

Calibration performance across included studies

Calibration was reported in seven of the 10 included studies using heterogeneous approaches and varying levels of detail [3,6,8,10,13-15]. Jeon et al. evaluated calibration using Brier scores and calibration plots and additionally applied isotonic regression, reporting improved agreement between predicted and observed probabilities after recalibration [3]. Li et al. also reported Brier scores and calibration plots, demonstrating acceptable prediction error and agreement across models [15]. Deng et al. assessed calibration using the Brier score, demonstrating lower prediction error in LSTM-based models compared with conventional logistic regression models [8].

Lin et al. evaluated calibration using calibration plots and the Hosmer-Lemeshow test, reporting no significant lack of fit [6]. Lim et al. assessed calibration using expected calibration error (ECE), demonstrating improved probability calibration with temporal modeling [10]. Pang et al. and Choi et al. presented calibration plots, which showed general agreement between predicted and observed outcomes without reporting quantitative measures [13,14]. In contrast, Mirzakhani et al., Huang et al., and Selcuk et al. did not report calibration assessments. Overall, calibration outcomes were variably described and not reported in a standardized or directly comparable format across studies [5,12,16]. Overall, calibration assessment methods varied across studies, with calibration plots, calibration slope, and Brier score being the most commonly reported measures. Several studies reported favorable calibration performance for AI-based models, although calibration reporting and validation approaches were inconsistent across studies.

Discussion

This systematic review evaluates the performance of AI-based models for mortality prediction in critically ill patients and compares them with conventional scoring systems, namely APACHE, SAPS, and SOFA. Overall, AI-based models have performed better than conventional scoring systems in terms of discrimination across various study settings, despite the identification of significant methodological limitations that could impact interpretation.

Across all the included studies, AI-based models showed high discriminative performance, with AUC values generally ranging from 0.83 to 0.95. These findings are in line with previous studies in the field of critical care. Wernly et al. demonstrated that LSTM-based deep learning models using routinely available arterial blood gas parameters could accurately predict mortality in ICU patients while outperforming conventional scoring approaches [23]. Similar gains in function have been reported in sepsis and critically ill populations in studies by Kong et al. and Zhang et al., where ML approaches outperformed conventional models by capturing complex clinical patterns [4,9]. Similarly, Guo et al. demonstrated strong survival prediction performance using ML and deep learning approaches in sepsis cohorts, supporting the broader applicability of AI-based prognostic models in critically ill populations [24]. In addition, recent reviews of biomarkers and sepsis prediction models support the broader role of data-driven and biomarker-informed approaches in improving mortality prediction [25,26].

Compared with conventional scoring systems, AI-based approaches demonstrated incremental gains in discriminative performance, with reported ΔAUC values ranging from +0.04 to +0.19. This degree of increment is clinically relevant and aligns with previous studies by van Doorn et al., Mirzakhani et al., Deng et al., Pang et al., and Choi et al., reporting similar incremental gains in predictive accuracy with ML approaches [2,5,8,13,14]. However, this comparison is based on descriptive synthesis rather than formal meta-analysis, because of substantial heterogeneity in study populations, differences in model development strategies, validation methodologies, outcome definitions, and reporting practices across included studies, limiting the application of meta-analysis. Furthermore, differences in cohort structure and the inclusion of multiple independently evaluated models within single studies limited direct statistical comparability. This reflects a broader limitation in the literature, where reporting of performance metrics remains inconsistent, as noted by Andaur Navarro et al. [27]. Methodological guidance from the Cochrane Handbook also acknowledges this limitation and notes that formal pooling may be unsuitable when effect estimates are not accompanied by corresponding measures of uncertainty, which was observed in several included studies [28]. Despite these limitations, the uniform direction of effect across all studies supports the conclusion that AI-based models generally perform better than conventional scoring systems such as APACHE, SOFA, and SAPS. Although the uniformly positive ΔAUC values across studies indicate a consistent direction of effect favoring AI-based models, this asymmetry may reflect publication or selective reporting bias. In the absence of a formal publication bias assessment, this potential bias should be considered when interpreting the findings.

Subgroup analyses demonstrated a progressive increase in pooled predictive performance from classical ML approaches to ensemble/tree-based models to deep learning architectures. Classical ML models demonstrated the lowest pooled AUC, whereas deep learning architectures achieved the highest pooled AUC. These findings suggest that advanced ML architectures may better capture nonlinear relationships and complex interactions within a heterogeneous ICU clinical setting. Tree-based and ensemble methods demonstrated strong predictive performance approaching close to that of deep learning models. Random forest, XGBoost, and LightGBM models consistently achieved high AUC values across several cohorts, indicating that sophisticated ensemble approaches remain highly competitive for ICU mortality prediction. These findings are consistent with previous studies by Pang et al. and Choi et al., who have reported a consistently better discriminative performance of ensemble and tree-based models compared with conventional statistical approaches using large-scale EHR datasets [13,14]. Ensemble methods may offer practical advantages, including less computational burden, better interpretability, and ease of implementation compared with more complex deep learning systems, while still maintaining strong predictive capability. However, the observed differences between subgroup estimates should be interpreted, considering important limitations like substantial heterogeneity across included cohorts, differences in study design, inconsistent validation methodologies, and the exploratory nature of subgroup analyses without formal statistical testing for subgroup interaction.

Interestingly, subgroup analysis based on temporal modeling demonstrated relatively similar pooled AUC values between longitudinal and static variable-based models. The finding contrasts with the growing body of literature suggesting potential advantages of temporal deep learning approaches in critical care prediction. Temporal models theoretically offer better prediction through the incorporation of sequential physiological trends, dynamic clinical trajectories, and continuously evolving patient states. Previous studies by Deng et al. demonstrated that LSTM-based frameworks were superior in mortality prediction by integrating temporal physiological information beyond conventional scoring systems [8]. Similarly, Lim et al. reported strong predictive performance of real-time ML systems using continuously updated ICU variables across international cohorts [10]. Komorowski et al. further highlighted the utility of reinforcement learning approaches for modeling evolving ICU trajectories and longitudinal patient states [29]. More recently, transformer-based architectures such as the Temporal Encoding Convolutional/Transformer-based Outcome model (TECO) model have demonstrated enhanced capability for capturing complex temporal dependencies within EHR data while maintaining robust external validation performance across diverse critical care populations [30]. Systematic evidence from Yan et al. supports the integration of longitudinal and time-dependent clinical variables for improving prediction performance in critical illness and sepsis-related outcomes [31]. Despite these theoretical and methodological advantages, temporal models in the present analysis did not demonstrate superior pooled AUC values compared with static approaches. Several factors may explain this observation. First, the static subgroup included highly optimized ensemble and boosting-based models, including random forest, XGBoost, and LightGBM frameworks, which already demonstrated strong discriminative performance using admission-based and summarized clinical variables. Second, many temporal models were evaluated using heterogeneous multicenter external validation cohorts, potentially reducing pooled performance estimates because of greater variability in patient populations, clinical workflows, and data structures. In contrast, studies evaluating static models were predominantly based on internally validated institutional cohorts, which may have contributed to comparatively higher pooled AUC values. Additionally, temporal modeling may improve clinically relevant aspects such as dynamic risk monitoring, repeated risk updating, and early deterioration detection without necessarily producing major increases in AUC, which primarily reflects overall discriminative ability rather than longitudinal responsiveness [29]. Collectively, these findings suggest that although temporal learning approaches remain promising, their incremental benefit over advanced static ensemble models may be smaller than theoretically expected when evaluated solely using pooled AUC metrics.

Dataset characteristics also appeared to influence predictive performance. Single-center cohorts demonstrated higher pooled AUC values compared with multicenter datasets. This observation may be explained by the influence of population heterogeneity, institutional practice patterns, data quality, and validation methodology. Single-center studies frequently relied on internally validated institutional datasets with relatively homogeneous patient populations and standardized workflows, conditions that may favor higher discriminative performance. In contrast, multicenter datasets in our study incorporated broader variability in ICU settings and patient characteristics, creating more challenging external validation environments. Previous studies by Lim et al. and Wernly et al. similarly emphasized the importance of multicenter validation for assessing the robustness and generalizability of AI-based ICU prediction models [10,23]. Supporting this observation, a recent large multicenter study by Zhao et al., involving over 107,000 ICU admissions across 15 hospitals, demonstrated that ML approaches significantly outperformed conventional scoring systems such as APACHE IV while maintaining strong calibration and temporal validation performance across diverse healthcare settings [32].

Considerable heterogeneity was observed across subgroup analyses, likely reflecting substantial methodological diversity between included studies, including differences in ICU populations, predictor selection, preprocessing strategies, model architectures, validation methods, and outcome definitions. Such heterogeneity in meta-analyses of ML prediction models highlights the challenges associated with comparing AI-based prognostic systems across diverse clinical environments. Emerging collaborative approaches such as federated learning may help improve external generalizability while enabling multi-institutional model development without centralized data sharing, as demonstrated by Vaid et al. in distributed EHR modeling studies [33]. In addition, broader advances in deep learning for EHR analysis have demonstrated an increasing ability to model complex sequential clinical patterns and improve predictive modeling across heterogeneous healthcare datasets [34]. Although these approaches were not developed specifically for ICU mortality prediction, they provide methodological support for improving generalizability and cross-institutional applicability of AI-based critical care prediction models.

A key strength of this study is the use of the PROBAST tool to assess risk of bias, which has been inconsistently applied in many of the previous systematic reviews of ML-based prediction models. The assessment demonstrated a generally low risk of bias in the participants, predictors, and outcome domains, while the analysis domain was found to be the main area of concern, largely due to incomplete reporting of model development, internal validation, and lack of calibration. These findings are in line with previous methodological studies. For example, Andaur Navarro et al. reported that many ML prediction models have important limitations in reporting and analysis, which can introduce bias and lead to overestimation of model performance [27]. By applying PROBAST, the present study was able to identify these issues more clearly and at a domain level.

In particular, gaps in validation and calibration are important, as they affect how well these models perform outside the original study setting. This may partly explain why reported performance is often high, but not always reproducible. Overall, greater transparency in reporting and stronger validation practices are needed in future studies. In this context, recently developed extensions such as TRIPOD-AI and PROBAST-AI (2024) provide more structured and AI-specific guidance for reporting and risk-of-bias assessment in prediction models [22,35]. Similarly, frameworks such as CONSORT-AI, SPIRIT-AI, and FUTURE-AI emphasize standardized reporting, prospective validation, and clinical evaluation, which are essential for improving reproducibility and facilitating the safe translation of AI systems into clinical practice [36-38].

Quantitative diagnostic test accuracy synthesis demonstrated favorable overall prognostic performance of AI-based models for mortality prediction in critically ill patients, with a pooled sensitivity of 0.845 and a pooled specificity of 0.791 derived from the Reitsma bivariate random-effects meta-analysis. The HSROC analysis further supported good overall discriminative performance across heterogeneous ICU populations. The relatively low random-effects correlation observed in the Reitsma model suggested the absence of a major threshold effect across included studies, indicating comparatively stable diagnostic discrimination despite variations in study design and AI architectures. Nevertheless, substantial heterogeneity was observed, particularly for specificity estimates, likely reflecting differences in patient populations, validation strategies, model complexity, outcome definitions, and reporting practices across studies. In addition, several studies did not report complete diagnostic contingency data directly, requiring reconstruction of 2×2 tables from reported performance metrics. These findings emphasize the need for more standardized reporting of diagnostic accuracy measures, including complete contingency data, external validation, calibration assessment, and transparent reporting of model development methodologies.

The findings of the present study are broadly comparable with prior systematic reviews. Barboi et al. reported wide variability in AI model performance and emphasized substantial heterogeneity and limited generalizability across studies [19]. Compared with previous analyses, the present study incorporates more recent deep learning and ensemble-based approaches and applies a structured methodological assessment using PROBAST. Overall, AI-based models demonstrated consistently improved discriminative performance compared with conventional scoring systems across included studies. Importantly, emerging literature suggests that successful translation of AI-based prediction systems into meaningful clinical benefit depends not only on predictive discrimination but also on integration into clinical workflows, interpretability, external validation, and real-world applicability [39].

From a clinical perspective, the favorable discriminative performance and sensitivity of AI-based models suggest potential utility in mortality prediction, early risk stratification, triage, and resource allocation among critically ill ICU patients. However, superior predictive performance alone does not necessarily translate into better patient outcomes [39]. Future studies should therefore focus not only on discrimination performance but also on prospective validation, calibration assessment, clinician interpretability, and real-world implementation within critical care workflows.

Limitations 

This study has several limitations. Formal quantitative pooling of AUC and ΔAUC values was not feasible due to the lack of variance or uncertainty measures required for robust meta-analysis. In addition, included studies variably reported internally validated and externally validated AUC estimates, introducing methodological heterogeneity that could potentially bias pooled discrimination estimates. Consequently, the comparative assessment of discriminative performance was primarily based on descriptive synthesis.

Substantial methodological and statistical heterogeneity was observed across studies included for quantitative analysis, both for specificity and sensitivity estimates. Differences in study design, ICU populations, AI model architectures, validation strategies, outcome definitions, and reporting practices may have influenced pooled estimates and limited generalizability. In addition, several studies did not directly report complete 2×2 contingency data, requiring reconstruction of TP, FP, FN, and TN values from reported performance metrics, which may have introduced estimation error. Calibration performance also could not be synthesized quantitatively because of inconsistent reporting across studies. The PROBAST assessment identified recurrent concerns within the analysis domain, particularly related to incomplete reporting of model development methodologies and external validation practices, which may affect reproducibility and generalizability of reported models. Finally, publication and selective reporting bias cannot be excluded, as studies reporting favorable AI model performance may have been more likely to be published.

## Conclusions

The present systematic review and meta-analysis was carried out to compare the performance of AI-based models with conventional scoring systems and demonstrated the overall better discriminative performance of AI-based models for predicting in-hospital mortality in critically ill patients. The observed performance advantage was particularly evident in deep learning and tree-based ensemble approaches, with temporal modeling strategies further supporting the potential value of dynamic longitudinal prediction in critical care settings. The risk of bias assessment indicated an overall acceptable methodological quality, with concerns primarily related to model validation and reporting. While pooled estimates suggest good overall prognostic accuracy, these findings should be interpreted in the context of substantial methodological and statistical heterogeneity across included studies.

Overall, AI-based approaches demonstrate promising prognostic performance for mortality prediction in critically ill patients; however, methodological heterogeneity, inconsistent reporting practices, and limited external validation across studies emphasize the need for further large-scale, standardized, and clinically integrated research before routine implementation in critical care settings.

## Appendices

Appendix A

Comprehensive Electronic Search Strategies for the Included Databases

Searches were conducted in PubMed, Embase, Scopus, and Web of Science for studies published between January 2015 and August 2025. Searches combined controlled vocabulary and free-text keywords related to critical illness, mortality, artificial intelligence/machine learning models, and conventional ICU scoring systems (APACHE, SOFA, SAPS) using Boolean operators and truncation. Filters applied across databases included human studies and English-language publications. The final literature search was completed on 30 November 2025. Search strategies were adapted according to the indexing syntax and controlled vocabulary of each database.

Database: PubMed

Search string: (("Intensive Care Units"[MeSH] OR "Critical Care"[MeSH] OR "critical illness"[tiab] OR "critically ill"[tiab] OR ICU[tiab] OR "intensive care"[tiab] OR "intensive care unit*"[tiab] OR "critical care unit*"[tiab]) AND ("Mortality"[MeSH] OR mortality[tiab] OR "in-hospital mortality"[tiab] OR "hospital mortality"[tiab] OR death[tiab] OR survival[tiab]) AND ("Artificial Intelligence"[MeSH] OR "Machine Learning"[MeSH] OR "Deep Learning"[tiab] OR "machine learning"[tiab] OR "artificial intelligence"[tiab] OR "neural network*"[tiab] OR "artificial neural network*"[tiab] OR "deep neural network*"[tiab] OR "random forest"[tiab] OR "gradient boosting"[tiab] OR XGBoost[tiab] OR LightGBM[tiab] OR "support vector machine"[tiab] OR SVM[tiab] OR "ensemble model*"[tiab] OR "predictive model*"[tiab] OR "prediction model*"[tiab] OR "prognostic model*"[tiab] OR "risk model*"[tiab]) AND (APACHE[tiab] OR "APACHE II"[tiab] OR "APACHE IV"[tiab] OR SOFA[tiab] OR "Sequential Organ Failure Assessment"[tiab] OR SAPS[tiab] OR "SAPS II"[tiab] OR "Simplified Acute Physiology Score"[tiab] OR "severity score"[tiab] OR "clinical score*"[tiab] OR "risk score*"[tiab]))

Database: Web of Science

Search string: TS=(("intensive care" NEAR/2 (unit* OR setting*) OR "critical care" NEAR/2 (unit* OR setting*) OR ICU OR "critical illness" OR "critically ill") AND (mortality OR "in-hospital mortality" OR "hospital mortality" OR death OR "survival rate*") AND ("artificial intelligence" OR "machine learning" OR "deep learning" OR "neural network*" OR "artificial neural network*" OR "deep neural network*" OR "artificial intelligence model*" OR "random forest" OR "gradient boosting" OR XGBoost OR LightGBM OR "support vector machine" OR SVM OR "ensemble model*" OR "predictive model*" OR "prediction model*" OR "prognostic model*" OR "risk model*") AND (APACHE OR "APACHE II" OR "APACHE IV" OR SOFA OR "Sequential Organ Failure Assessment" OR SAPS OR "SAPS II" OR "Simplified Acute Physiology Score" OR "severity score" OR "clinical score*" OR "risk score*"))

Database: Embase

Search string: ('intensive care unit'/exp OR 'critical care'/exp OR 'critical illness'/exp OR 'intensive care':ti,ab OR 'intensive care unit*':ti,ab OR ICU:ti,ab OR 'critical care':ti,ab OR 'critical care unit*':ti,ab OR 'critical illness':ti,ab OR 'critically ill':ti,ab) AND ('mortality'/exp OR mortality:ti,ab OR 'in-hospital mortality':ti,ab OR 'hospital mortality':ti,ab OR death:ti,ab OR 'survival rate*':ti,ab) AND ('artificial intelligence'/exp OR 'machine learning'/exp OR 'deep learning'/exp OR 'neural network'/exp OR 'artificial intelligence':ti,ab OR 'machine learning':ti,ab OR 'deep learning':ti,ab OR 'neural network*':ti,ab OR 'artificial neural network*':ti,ab OR 'deep neural network*':ti,ab OR 'random forest':ti,ab OR 'gradient boosting':ti,ab OR xgboost:ti,ab OR lightgbm:ti,ab OR 'support vector machine':ti,ab OR svm:ti,ab OR 'ensemble model*':ti,ab OR 'predictive model*':ti,ab OR 'prediction model*':ti,ab OR 'prognostic model*':ti,ab OR 'risk model*':ti,ab) AND (apache:ti,ab OR 'apache ii':ti,ab OR 'apache iv':ti,ab OR sofa:ti,ab OR 'sequential organ failure assessment':ti,ab OR saps:ti,ab OR 'saps ii':ti,ab OR 'simplified acute physiology score':ti,ab OR 'severity score':ti,ab OR 'clinical score*':ti,ab OR 'risk score*':ti,ab) AND [humans]/lim AND [english]/lim

Database: Scopus

Search string: TITLE-ABS-KEY(("intensive care" OR "intensive care unit*" OR ICU OR "critical care" OR "critical care unit*" OR "critical illness" OR "critically ill") AND (mortality OR "in-hospital mortality" OR "hospital mortality" OR death OR "survival rate*") AND ("artificial intelligence" OR "machine learning" OR "deep learning" OR "neural network*" OR "artificial neural network*" OR "deep neural network*" OR "random forest" OR "gradient boosting" OR xgboost OR lightgbm OR "support vector machine" OR svm OR "ensemble model*" OR "predictive model*" OR "prediction model*" OR "prognostic model*" OR "risk model*") AND (apache OR "apache ii" OR "apache iv" OR sofa OR "sequential organ failure assessment" OR saps OR "saps ii" OR "simplified acute physiology score" OR "severity score" OR "clinical score*" OR "risk score*"))

Appendix B

PROBAST Risk-of-Bias Assessment Framework

Risk-of-bias assessment was conducted using the PROBAST tool. Assessments were performed across the domains of participants, predictors, outcomes, and analysis. Responses to signaling questions were categorized as Yes (Y), Probably Yes (PY), Probably No (PN), No (N), or No Information (NI), followed by domain-level and overall risk-of-bias judgments.

Standard PROBAST Risk of Bias Assessment Form

Review Question

Intended use:

Participants:

Predictors:

Outcome:

Study Information

Type of model (Development / Validation / Both):

Outcome assessed:

DOMAIN 1: Participants

Appropriate data sources

Inclusion/exclusion criteria

Risk-of-bias judgment

Applicability concern

DOMAIN 2: Predictors

Predictor definition and consistency

Blinded assessment of predictors

Availability at time of use

Risk-of-bias judgment

Applicability concern

DOMAIN 3: Outcome

Outcome definition and assessment

Consistency of outcome measurement

Predictor exclusion from outcome definition

Appropriate time interval

Risk-of-bias judgment

Applicability concern

DOMAIN 4: Analysis

Sample size adequacy

Missing data handling

Participant inclusion

Overfitting assessment

Performance evaluation

Risk-of-bias judgment

Applicability concern

Overall Assessment

Overall risk of bias

Overall applicability concern

Summary rationale

Appendix C

Standardized Data Extraction Framework

A representative standardized data extraction template is provided below. The complete operational extraction form used during the review process contained additional study-specific and methodological fields; however, the present template includes the principal variables relevant to the study (Table 9).

**Table 9:** Summary of variables included in the standardized data extraction form.

Domain	Variables extracted
Study characteristics	First three authors, publication year, country/region, study design, study setting (single-center/multicenter), ICU type, dataset source, study period
Population characteristics	Total sample size, mortality events/deaths, survivors/non-events, mortality prevalence, inclusion criteria, exclusion criteria
Outcome measures	In-hospital mortality and ICU mortality
AI model characteristics	AI/ML model evaluated, best-performing model, model category (classical ML/tree-based/deep learning/ensemble), temporal modelling use, number of predictors/features, validation type (internal/external), training and validation cohort size
Comparator characteristics	APACHE, SOFA, SAPS scoring systems.
Performance metrics	AUC, 95% confidence intervals, sensitivity, specificity, accuracy, calibration method, TP, FP, FN, TN values when available.

Appendix D

Statistical Analysis Workflow and Representative R Code

Statistical analyses were performed in R using the meta, metafor, and mada packages. Exploratory subgroup analyses were conducted using inverse variance-weighted random-effects meta-analysis with the DerSimonian-Laird estimator for pooled AUC estimates across predefined subgroup categories. Diagnostic test accuracy synthesis of sensitivity and specificity was performed using the Reitsma bivariate random-effects model based on 2×2 contingency table data.

Representative analytical workflows used for subgroup analyses and diagnostic test accuracy synthesis are summarized below.

Exploratory subgroup analysis (DerSimonian-Laird random-effects model)

library(meta)

meta_subgroup <- metagen(

TE = AUC,

seTE = SE,

studlab = Study,

data = dataset,

sm = "SMD",

method.tau = "DL",

hakn = FALSE

)

forest(meta_subgroup)

...

Note: The same analytical framework was applied for subgroup analyses based on AI model type, dataset characteristics, and temporal modeling approach.

Diagnostic test accuracy meta-analysis (Reitsma bivariate model)

library(mada)

dta_data <- data.frame(TP, FN, FP, TN)

reitsma_model <- reitsma(dta_data)

summary(reitsma_model)

plot(reitsma_model,

     sroclwd = 2,

     main = "HSROC Curve")

...

Appendix E

Comparison Between Prespecified PROSPERO Protocol Items and the Final Review Methodology

The review methodology remained broadly consistent with the prospectively registered PROSPERO protocol. Minor methodological refinements and exploratory analyses were introduced during the review process because of variability in study design, inconsistent reporting of performance metrics, and heterogeneity in AI model architectures (Table 10).

**Table 10:** Comparison between prespecified PROSPERO protocol items and final conducted review methodology.

Protocol item	Final approach in study	Remarks
Mortality outcome	ICU mortality was accepted as a surrogate outcome when in-hospital mortality was unavailable.	Outcome reporting variability across studies
PROBAST assessment	Conducted using a domain-level assessment	Domain-wise assessments are summarized in the manuscript.
Subgroup analyses	Planned: AI modality, ICU type, and geographic region. Conducted: AI model type; dataset characteristics (single-center vs multicenter); and temporal modeling analyses.	ICU setting and geographic region analyses were limited by small subgroup sizes, inconsistent reporting, and insufficient homogeneous study-level data. Additional exploratory subgroup analyses were conducted to further assess heterogeneity.
Calibration analysis	Calibration metrics were synthesized descriptively.	Quantitative pooling was not feasible because of heterogeneous calibration reporting methods.
Diagnostic test accuracy synthesis	Performed using directly reported or derivable contingency data.	Due to limited direct reporting of contingency data, only eight studies were eligible for quantitative diagnostic test accuracy synthesis.
